# Supplementary material for: A single day of TGF-β1 exposure activates chondrogenic and hypertrophic differentiation pathways in bone marrow-derived stromal cells
Source: Commun Biol. 2021 Jan 4;4:29. doi: 10.1038/s42003-020-01520-0 (PMC7782775; doi:10.1038/s42003-020-01520-0)
Supplement: Supplementary file 1 — Description of Supplementary Files [file 42003_2020_1520_MOESM1_ESM.pdf]

Description of Additional Supplementary Files

File Name: Supplementary Data 1.

Description: Differentially expressed genes between BMSC and ACh. Positive sign represents higher expression in ACh relative to BMSC. Negative sign represents higher expression in BMSC relative to ACh.

File Name: Supplementary Video 1.

Description: Animation of Microwell-mesh cell seeding. This animation demonstrates how the Microwell-mesh can be used to efficiently manufacture hundreds of micro-pellets. The Microwell-mesh is a microwell platform with a nylon mesh bound over the microwells. This platform can be seeded with cells by centrifuging a cell suspension through the mesh, and into the microwells. Single cell suspensions easily pass through the mesh which has 36 micron square openings. When the cells in the microwells aggregate into micro-pellets, the micro-pellets are too large to pass back through the mesh, and thus remain trapped within discrete microwells. This novel feature of the Microwell-mesh allows it to retain micro-pellets in discrete microwells over complex cultures, including multiple media exchanges.
