# Supplementary material for: A single day of TGF-β1 exposure activates chondrogenic and hypertrophic differentiation pathways in bone marrow-derived stromal cells
Source: Commun Biol. 2021 Jan 4;4:29. doi: 10.1038/s42003-020-01520-0 (PMC7782775; doi:10.1038/s42003-020-01520-0)
Supplement: Supplementary file 4 — Reporting Summary [file 42003_2020_1520_MOESM4_ESM.pdf]

nature research

Corresponding author(s): Michael Robert Doran

Last updated by author(s): November 18, 2020

Reporting Summary

Nature Research wishes to improve the reproducibility of the work that we publish. This form provides structure for consistency and transparency in reporting. For further information on Nature Research policies, see our Editorial Policies and the Editorial Policy Checklist.

Statistics

For all statistical analyses, confirm that the following items are present in the figure legend, table legend, main text, or Methods section.

n/a	Confirmed	
☐	☒	The exact sample size (*n*) for each experimental group/condition, given as a discrete number and unit of measurement
☐	☒	A statement on whether measurements were taken from distinct samples or whether the same sample was measured repeatedly
☐	☒	The statistical test(s) used AND whether they are one- or two-sided<br>*Only common tests should be described solely by name; describe more complex techniques in the Methods section.*
☒	☐	A description of all covariates tested
☐	☒	A description of any assumptions or corrections, such as tests of normality and adjustment for multiple comparisons
☐	☒	A full description of the statistical parameters including central tendency (e.g. means) or other basic estimates (e.g. regression coefficient) AND variation (e.g. standard deviation) or associated estimates of uncertainty (e.g. confidence intervals)
☒	☐	For null hypothesis testing, the test statistic (e.g. *F*, *t*, *r*) with confidence intervals, effect sizes, degrees of freedom and *P* value noted<br>*Give P values as exact values whenever suitable.*
☒	☐	For Bayesian analysis, information on the choice of priors and Markov chain Monte Carlo settings
☒	☐	For hierarchical and complex designs, identification of the appropriate level for tests and full reporting of outcomes
☒	☐	Estimates of effect sizes (e.g. Cohen's *d*, Pearson's *r*), indicating how they were calculated

Our web collection on statistics for biologists contains articles on many of the points above.

Software and code

Policy information about availability of computer code

Data collection	NA
Data analysis	ANOVA and subsequent graphing using Graphpad Prism (version 7). RNA-Seq data was analyzed with Bioconductor R package edgeR. Heatmaps were generated using the heatmap.2 function.

For manuscripts utilizing custom algorithms or software that are central to the research but not yet described in published literature, software must be made available to editors and reviewers. We strongly encourage code deposition in a community repository (e.g. GitHub). See the Nature Research guidelines for submitting code & software for further information.

Data

Policy information about availability of data

All manuscripts must include a data availability statement. This statement should provide the following information, where applicable:

- Accession codes, unique identifiers, or web links for publicly available datasets
- A list of figures that have associated raw data
- A description of any restrictions on data availability

Data supporting the conclusions of this paper are available from the corresponding author upon request. Raw RNA-seq data has been uploaded to the NCBI database, and can be found here: GSE161176: https://urldefense.com/v3/__https://www.ncbi.nlm.nih.gov/geo/query/acc.cgi?acc=GSE161176__;!!NVzLfOphnbDXSw!T6T9I2u7Cag6HE_W7hGM8VnUtIahLNPw6oAvC5QpjicFhRSmoZBfTl-u0Czs3HMuVuOe1A$

Field-specific reporting

Please select the one below that is the best fit for your research. If you are not sure, read the appropriate sections before making your selection.

- [x] Life sciences
- [ ] Behavioural & social sciences
- [ ] Ecological, evolutionary & environmental sciences

For a reference copy of the document with all sections, see nature.com/documents/nr-reporting-summary-flat.pdf

Life sciences study design

All studies must disclose on these points even when the disclosure is negative.

Sample size	We indicate the number of BMSC and ACh donors and replicates in each figure caption, and in the text. The primary comparison of BMSC and ACh, were completed using 4 BMSC donors and 2 ACh donors. Each experiment had 4-6 replicate cultures.
Data exclusions	We did not exclude data.
Replication	As above, the primary studies were replicated with 4 BMSC donors and 2 ACh donors.
Randomization	We did not randomise experiments.
Blinding	We did not blind experiments.

Reporting for specific materials, systems and methods

We require information from authors about some types of materials, experimental systems and methods used in many studies. Here, indicate whether each material, system or method listed is relevant to your study. If you are not sure if a list item applies to your research, read the appropriate section before selecting a response.

Materials & experimental systems

n/a	Involved in the study	
x		Antibodies
	x	Eukaryotic cell lines
x		Palaeontology and archaeology
	x	Animals and other organisms
x		Human research participants
x		Clinical data
x		Dual use research of concern

Methods

n/a	Involved in the study	
x		ChIP-seq
x		Flow cytometry
x		MRI-based neuroimaging

Eukaryotic cell lines

Policy information about cell lines

Cell line source(s)	We derived our own BMSC from bone marrow aspirates. We purchased ACh from Lonza.
Authentication	Not completed.
Mycoplasma contamination	Not completed.
Commonly misidentified lines (See ICLAC register)	NA

Animals and other organisms

Policy information about studies involving animals; ARRIVE guidelines recommended for reporting animal research

Laboratory animals	NSG mice were used in this study. We used both sexes (predominantly female), at approximately 6-12 weeks of age.
Wild animals	NA
Field-collected samples	NA
Ethics oversight	The University of Queensland (UQ) and the Queensland University of Technology (QUT) Animal Ethics Committees reviewed and approved the animal ethics applications.

Note that full information on the approval of the study protocol must also be provided in the manuscript.
